# The Role of Serum Adiponectin for Outcome Prediction in Patients with Dilated Cardiomyopathy and Advanced Heart Failure

**DOI:** 10.1155/2017/3818292

**Published:** 2017-11-26

**Authors:** Vaida Baltrūnienė, Daiva Bironaitė, Ieva Kažukauskienė, Julius Bogomolovas, Dalius Vitkus, Kęstutis Ručinskas, Edvardas Žurauskas, Renaldas Augulis, Virginija Grabauskienė

**Affiliations:** ^1^Department of Pathology, Forensic Medicine and Pharmacology, Faculty of Medicine, Vilnius University, M. K. Ciurlionio 21, LT-03101 Vilnius, Lithuania; ^2^Department of Regenerative Medicine, State Research Institute, Center for Innovative Medicine, Santariskiu 5, LT-08406 Vilnius, Lithuania; ^3^Department of Cardiology and Angiology, Vilnius University Hospital Santaros Klinikos, Santariskiu 2, LT-08661 Vilnius, Lithuania; ^4^Department of Integrative Pathophysiology, Medical Faculty Mannheim, Mannheim, Germany; ^5^Department of Physiology, Biochemistry, Microbiology and Laboratory Medicine, Faculty of Medicine, Vilnius University, Vilnius, Lithuania

## Abstract

Clinical interpretation of patients' plasma adiponectin (APN) remains challenging; its value as biomarker in dilated cardiomyopathy (DCM) is equivocal. We evaluated whether circulating APN level is an independent predictor of composite outcome: death, left ventricle assist device (LVAD) implantation, and heart transplantation (HT) in patients with nonischemic DCM. 57 patients with nonischemic DCM (average LV diastolic diameter 6.85 cm, LV ejection fraction 26.63%, and pulmonary capillary wedge pressure 22.06  mmHg) were enrolled. Patients underwent echocardiography, right heart catheterization, and endomyocardial biopsy. During a mean follow-up of 33.42 months, 15 (26%) patients died, 12 (21%) patients underwent HT, and 8 (14%) patients were implanted with LVAD. APN level was significantly higher in patients who experienced study endpoints (23.4 versus 10.9 ug/ml, *p* = 0.01). APN was associated with worse outcome in univariate Cox proportional hazards model (HR 1.04, CI 1.02–1.07, *p* = 0.001) but lost significance adjusting for other covariates. Average global strain (AGS) is an independent outcome predictor (HR 1.42, CI 1.081–1.866, *p* = 0.012). Increased circulating APN level was associated with higher mortality and may be an additive prognostic marker in DCM with advanced HF. Combination of serum (APN, BNP, TNF-*α*) and echocardiographic (AGS) markers may increase the HF predicting power for the nonischemic DCM patients.

## 1. Introduction

In recent years, the concept of chronic heart failure (CHF) pathogenesis has changed dramatically. It became clear that CHF is not simply a hemodynamic failure and even not a problem of impaired neuroendocrine activation; it is a far more complex process, a systemic disorder, which involves immune activation, metabolic alterations, and pathologic processes in skeletal muscle [1].

Adiponectin (APN) is an adipocyte-derived cytokine (adipokine), which is also synthesized in cardiac muscle cells and connective tissue cells within the heart [2]. APN has a critical signaling function in the heart which is particularly important in patients with heart failure.

Its beneficial cardioprotective effects leave no doubt. APN has antiapoptotic, fibrosis reducing, and oxidative stress diminishing properties in myocardium [3, 4]. Lower serum APN is an independent cardiovascular risk factor in coronary artery disease [5–8]. Low serum APN levels also increase cardiovascular risk and inflammation in hypertension, coronary artery disease, obesity, and insulin resistance [5, 9, 10] and correlates with left ventricular (LV) hypertrophy [11, 12]. On the other hand, high APN levels are associated with increased risk of recurrent cardiovascular events [13] and mortality in patients with acute myocardial infarction [14] and heart failure [15, 16]. In patients with systolic HF, APN levels are increased and correlate with mortality, disease severity, and HF symptoms [17, 18]. Adiponectin concentration increases with increasing HF severity and parallels NYHA functional class [19–22]. It is still a question of debate whether APN loses its cardioprotective function in CHF or it fails to control progression of disease.

APN has also been investigated as a prognostic marker in CHF. Although it seems that APN has an additive role in predicting the disease course [23–25], it has not been officially recognized as a biomarker in HF with reduced ejection fraction [26].

Tamura and coauthors show that APN can be an independent predictor of mortality in patients with ischemic CHF. But they did not find significant impact of high serum APN level on the mortality of patients with nonischemic CHF [25].

Nonischemic DCM is an important cause of HF and heart transplantation (HT). So there is a compelling need for markers predicting the prognosis and disease course of the end-stage heart failure caused by DCM. It is also of great importance in prioritizing patients' list for transplantation.

In this study, we investigated the predictive potential of serum APN with regard to LVAD implantation, HT, and mortality in a cohort of patients with nonischemic dilated DCM and advanced HF and analyzed the associations between APN and other biomarkers of CHF.

## 2. Material and Methods

### 2.1. Patients

Our study cohort was composed of patients admitted to Vilnius University Hospital “Santaros klinikos” with suspected diagnosis of idiopathic DCM. The patient inclusion criteria were exacerbation of heart failure symptoms, accompanied by LV dilation, reduced LV ejection fraction (LVEF < 45%) and the absence of significant coronary artery disease (stenosis of coronary arteries of more than 50%), a history of myocardial infarction, and other specific heart muscle diseases (primary valvular heart disease, toxic cardiomyopathy, arterial hypertension, renal failure, and abuse of alcohol or illicit drugs).

All patients underwent a careful history and physical examination, routine laboratory studies, including high-sensitivity C reactive protein (CRP), brain natriuretic peptide (BNP), adiponectin, tumor necrosis *α* (TNF-*α*), interleukin-6 (IL-6), and cardiac troponin T (hsTnT) as well as echocardiography to evaluate LV function and EF. NYHA class was assigned prior to echocardiographic investigation. Mandatory investigations included coronary angiography to exclude significant coronary disease, right heart catheterization for hemodynamic evaluation, and EMB for evaluation of inflammatory infiltrates in myocardium.

57 patients with nonischemic DCM (average LV end diastolic diameter (LVEDD) 6.85 ± 0.86 cm, LVEF 26.4 ± 9.45%, and mean pulmonary capillary wedge pressure (PCWP) 22.06 ± 8.97 mmHg) were enrolled into the study consistent with primary DCM. 49 (90%) of the patients were ranked as NYHA III and IV classes and all had increased BNP values.

All patients had long duration of HF symptoms: at enrollment, the average duration of observed symptoms was 40 ± 53 months.

At the moment of enrollment to the study, the patients were normotensive to hypotensive: the average of systolic blood pressure was 116 ± 20 mmHg and average of diastolic pressure was 80 ± 10 mmHg. Patients with long standing arterial hypertension in anamnesis were not included in this study. Patients diagnosed with diabetes were not enrolled.

All patients received pharmacological HF therapy according to the guidelines of European Society of Cardiology [27]: ACE inhibitors or blockers of angiotensin receptors, *β*-blockers, mineralocorticoid receptors blockers, digitalis (in case of atrial fibrillation), diuretics, anticoagulant (in case of atrial fibrillation, LVEF < 40%), and antiarrhythmic (class III: amiodarone). Thiazolidinedione was not administered to any patients at the time of blood sample collection. Clinical decision regarding cardiac resynchronization therapy, radiofrequency ablation, implantation of LVAD, or cardioverter-defibrillator was made after coronary angiography and right heart catheterization. In case of histologically proven acute myocarditis, patients were excluded from the study. Baseline characteristics of all patients are presented in Tables 1 and 2.

### 2.2. Follow-Up Period

Patients were followed up for a mean of 33.42 ± 21 months. The first date of the follow-up was the date of taking EMB. The endpoint of this study was composite and combined three possible outcomes: death from cardiovascular causes, LVAD implantation, or HT. The rationale to use this composite end point was that all those states meant either death or a very severe cardiac state of the patient with exhausted therapeutic measures. Some of the patients experienced several outcomes. The time of the endpoint was the time of the first event.

During the follow-up period, 25 patients (43.8%) reached endpoint of the study (died or underwent HT or LVAD implantation). Patients were subdivided into two groups according to their outcomes: those who have reached follow-up endpoint *n* = 25 and the ones who did not – *n* = 32 in order to see if there is a significant difference in level of APN in both groups.

All deaths and other endpoint outcomes were confirmed by medical records or telephone interview with the patients' families.

### 2.3. Biochemical Assays of APN and Other Serological Markers

Blood samples data were obtained shortly after admission. Blood was drawn at the same day as cardiac catheterization.

The proinflammatory serum cytokines TNF-*α* and IL-6 were measured by solid-phase, chemiluminescent immunometric assays using IMMULITE/Immulite 1000 systems (Immulite, Siemens) according to manufactures instructions: TNF-*α* (Catalog number LKNFZ (50 tests) and LKNF1 (100 tests)), IL-6 (Catalog number LK6PZ (50 tests) and LK6P1 (100 tests)). Adiponectin was measured by Millipore Adiponectin assay according to manufacturers' recommendations (Millipore, USA).

The myocardial necrosis marker, high-sensitivity troponin T (hsTnT), was measured in serum using an Elecsys 2010 analyzer (Roche Diagnostics, Indianapolis, Indiana) and expressed as pg/ml.

Brain natriuretic peptide (BNP) was measured by a two-step immunoassay in human plasma using CMIA technology and protocols referred as Chemiflex. Briefly, sample and anti-BNP coated paramagnetic particles were combined. After incubation, samples were washed and combined with an anti-BNP acridinium-labeled conjugate. Samples were incubated and washed again and the chemoluminescence initiating mixture was added. Resulting chemiluminescent reaction was measured by chemiluminometer and expressed as relative light units (RLU).

### 2.4. Echocardiography

Echocardiographic evaluation was accomplished on admission by investigator blinded for the study objectives. GE Vivid 7 and 9 ultrasound systems were used. The standard LV apical (apical 4, apical 2, and apical 3) views and parasternal short axis views at mid-papillary level were acquired at 70–90 frames/s. Conventional echocardiographic parameters such as LVEF, LVEDD, left ventricular end-systolic dimension (LVESD) [28], velocities of E and A waves (E and A) and their ratio (E/A), and E deceleration time (DcT) were obtained. All images were stored in PACS for subsequent analysis. Quantification of myocardial deformation values was performed by 2D speckle tracking using Echopac PCBT08 (GE Healthcare) software. After the manual selection, speckles were assumed automatically and then confirmed by the investigator. Longitudinal, circumferential, and radial strain and strain rate parameters were extracted using semiautomatic postprocessing. Global strain is presented as the mean value of all valid segments. Global strain in our measurements showed an interobserver variability of 1.1 + 0.9% and an interobserver variability of 1.3 + 1.2%.

### 2.5. Cardiac Catheterization and Endomyocardial Biopsy

All patients signed written informed consent for cardiac catheterization and EMB and coronary angiography, which included resulting analysis to elucidate a possible origin of the myocardial and coronary artery diseases. Each patient underwent coronary angiography to exclude significant coronary artery disease (stenosis > 50%) and right heart catheterization to assess hemodynamic parameters: mean pulmonary artery pressure (PAP), right atrial pressure (RAP), pulmonary capillary wedge pressure (PCWP), and cardiac output (CO).

Right ventricular EMB was obtained using a flexible bioptome* (Westmed)* via the right femoral vein [29]. Biopsies were drawn from the right interventricular septum. At least 3 EMBs were subjected to conventional histologic and immunohistochemical evaluation and 2 EMBs were stored at −70 C in the biobank as retained biosamples. EMBs were immediately placed on ice and investigated within 24 hours.

### 2.6. Histological and Immunohistochemical Assessment of EMBs

EMB samples for histological analysis were fixed in 10% buffered formalin and subsequently paraffin-embedded in a tissue processor. 3 *μ*m thick sections were used through the study. The EBM sections were stained with Hematoxylin and Eosin (H&E) according to the standard protocol for the routine histological evaluation. Histological diagnosis was based on the Dallas criteria [30, 31]. The experienced pathologist evaluated endocardium (thickness, subendocardial fat, fibrosis, and inflammation); myocardium (muscle fiber number, size, and damage); interstitium (fibrosis, fat, edema, and inflammation); and intramural vessels (size, signs of inflammation, damage, and luminal stenosis). Immunohistological assessment of EMBs was carried out as described elsewhere [32]. Autoantibodies (Santa Cruz Biotechnology, Inc.) against CD3+ (DAKO A0452 Rabbit 1, Hamburg, Germany), CD45Ro (DAKO Hamburg), and CD68+ (DAKO M0876 Mouse 1, Hamburg) were used for immunohistochemical staining. The number of positively stained cells in each biopsy sample was scored by an experienced pathologist and expressed as number of positive cells/mm2. EMB were considered to be inflamed if IHC staining revealed significant inflammatory cellular infiltrates (≥14 leucocytes/mm2 including up to 4 monocytes/mm2 with the presence of CD3 positive T-lymphocytes ≥7 cells/mm2) [33, 34].

### 2.7. Statistical Analysis

Statistical analyses were performed using the SPSS package (version 23.0 for Windows; IBM.SPSS statistics) and R studio package (version 1.0.143 – © 2009–2016 RStudio, Inc.) at not higher than 5% significance level. The normality of the data distribution was tested by the Shapiro-Wilk test. Variables which did not follow normal distribution were expressed as medians (interquartile ranges). All the other continuous variables were expressed as means ± SD.

Significance of measurements was tested by Student's* t*-test or the Wilcoxon–Mann–Whitney rank sum nonparametric test.

For comparative purposes, Spearman's correlation coefficient was used. Testing the differences between parameter values in the subgroups of nonischemic DCM patients (good versus bad outcome groups, high versus low adiponectin groups) Student's* t*-test or the nonparametric Mann–Whitney* U* test (serum adiponectin, BNP, IL-6, TNF-*α*, TnT, CRP, etc.) was used. Differences between categorical variables were tested using Chi-squared test.

Kaplan–Meier analysis was used to compare the cumulative survival rates between the 2 subgroups of nonischemic DCM patients stratified according to the median serum APN levels. Differences between the survival times were tested using a log-rank analysis for APN. Univariate analysis with the Cox proportional hazards model was used to assess the association of each variable with patient survival. Multivariate analysis with the Cox proportional hazards model was used to assess the independence of the predictors of composite endpoint. The covariates included (1) the parameters with *p* < 0.05 in the univariate analysis (APN, IL-6, average global strain, CD3+ cell number in myocardium, and PCWP) and (2) the established predictors of mortality in CHF patients (gender, age, EF, NYHA class IV versus I–III, and GFR). Stepwise selection procedure was used for choosing the independent predictors of outcome.

For the search of a set of variables which could be a reflection of some more global parameter or be a good combination for predicting patient outcome factor analysis was used.

### 2.8. Ethical Approval

The study was approved by the local Lithuanian Bioethics Committee (license numbers 158200-09-382-l03; 158200-382-PP1-23; and 158200-17-891-413). Informed consent was signed by all study patients. Our study was conducted in accordance with the Declaration of Helsinki.

## 3. Results

### 3.1. Correlations between Serum Adiponectin Level, Cardiac Hemodynamics, and Inflammation in Patients with Nonischemic DCM

**Figure 1:**
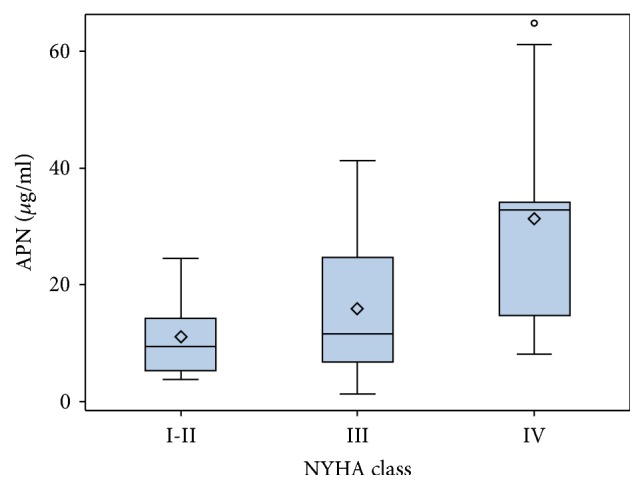
Adiponectin according to NYHA classes.

The median of adiponectin in our cohort of patients was 14.2 ± 20.8 *μ*g/ml. There was a tendency for APN concentration to rise in each subsequent NYHA functional class. (Figure 1). APN concentration was statistically significantly higher in NYHA functional class IV in comparison to NYHA functional class III (*p* = 0.014). We found no difference in APN means between I + II and III NYHA functional classes (*p* = 0.712).

**Figure 2:**
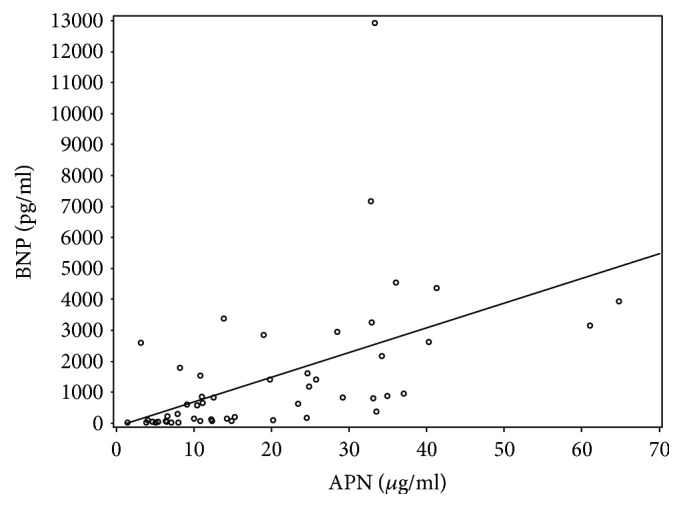
APN correlation with BNP.

**Figure 3:**
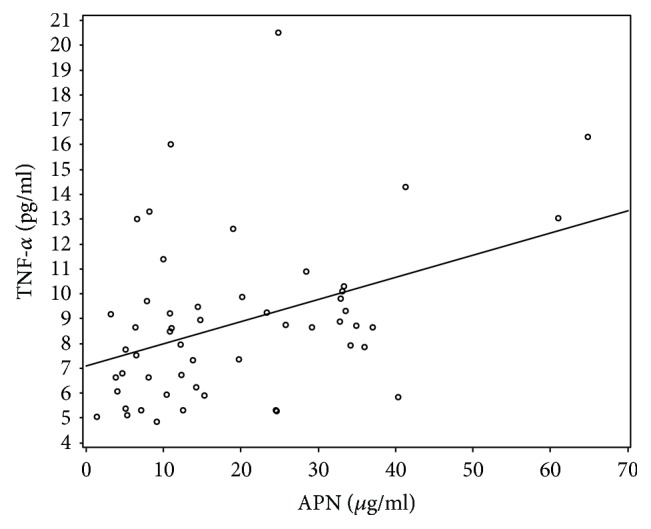
APN correlation with TNF-*α*.

The significant correlation between APN and BNP (rho 0.65, *p* = 0.001) was found and is shown in (Figure 2). APN has also had a positive correlation with inflammatory cytokine TNF-*α* (rho 0.331, *p* = 0.021) (Figure 3), although correlation with another inflammatory cytokine IL-6 (rho 0.257, *p* = 0.058) was not statistically significant. Both inflammatory cytokines IL-6 (rho 0.656, *p* < 0.001) and TNF-*α* (rho 0.504, *p* = 0.004) significantly correlated with BNP.

**Figure 4:**
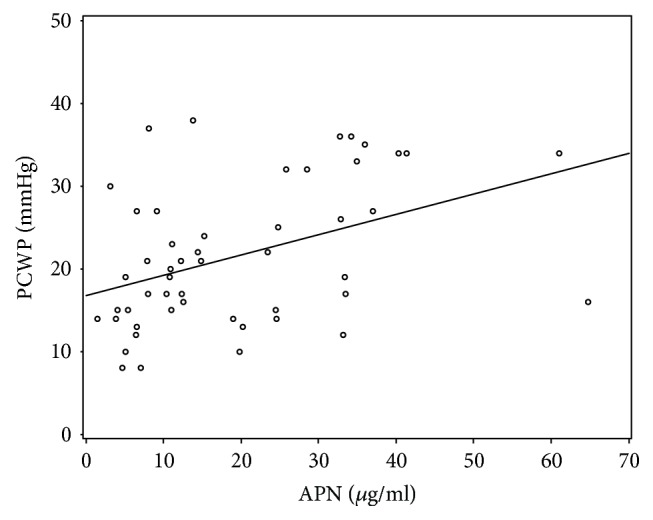
APN correlation with PCWP.

**Figure 5:**
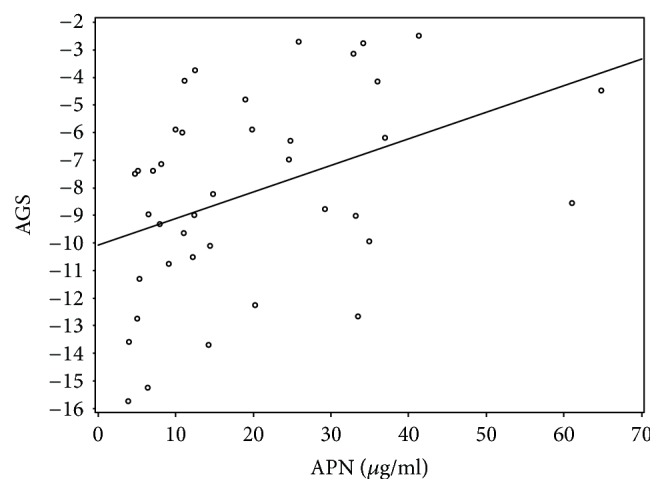
APN correlation with average global strain (AGS).

APN also positively correlated with certain hemodynamic parameters such as mean PCWP (rho 0.38, *p* = 0.005) (Figure 4) and mean PAP (rho 0.434, *p* = 0.001). There was a significant association between APN and LV dysfunction parameter, average global strain (rho 0.472, *p* = 0.002) (Figure 5). We found no significant correlation with LVEF and negative correlation with BMI did not reach statistical significance (*p* = 0.054). APN correlation data are shown in Table 3.

### 3.2. Adiponectin Level Is Significantly Higher in the Bad Outcome Group

During the follow-up period, 25 patients (43.8%) reached endpoint of the study: 15 (26%) patients died because of cardiovascular causes, 12 (21%) patients underwent heart transplantation (HT), and 8 (14%) were implanted with LVAD. 3 out of 8 patients with LVAD underwent HT later on.

**Figure 6:**
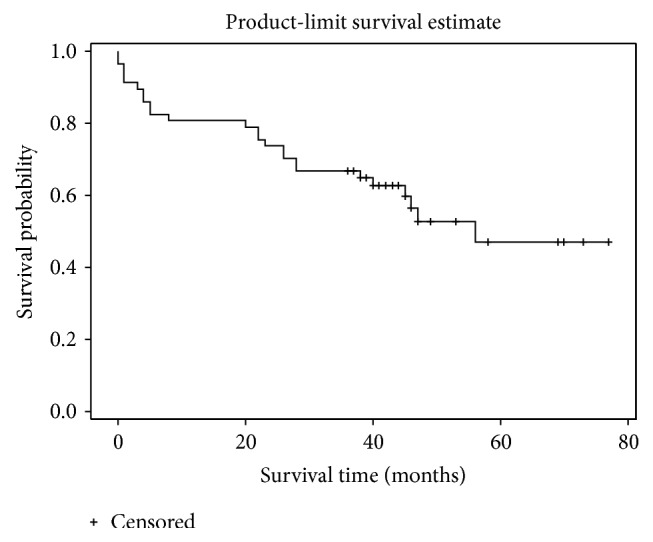
Kaplan–Meier survival curve for the whole study cohort.

Kaplan–Meier cumulative survival curve was drawn Figure 6.

**Figure 7:**
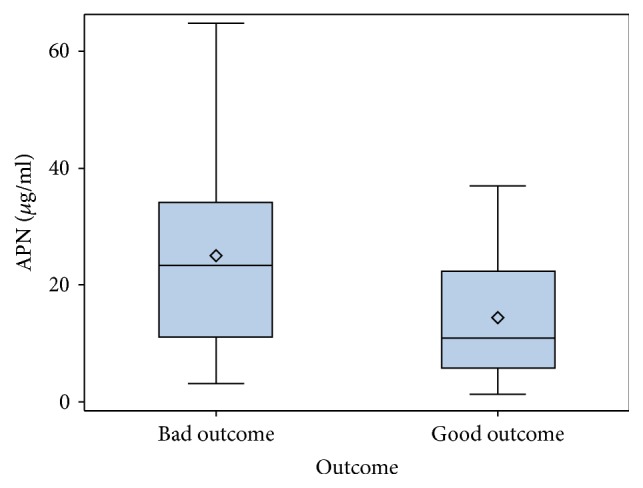
Adiponectin level in good and bad outcome groups.

The patients were divided into two groups according to their outcome: bad outcome (the ones who reached the composite endpoint) *n* = 25 and good outcome group *n* = 32. The groups did not differ in their age (*p* = 0.08), sex (*p* = 0.863), NYHA class (*p* = 0.119), BMI (*p* = 0.51), and GFR (*p* = 0.30) (Table 4). There was significant difference in the baseline concentration of APN between the two groups (10.9 ± 17.87 *μ*g/ml versus. 23.4 ± 23.1 *μ*g/ml, *p* = 0.01) (Figure 7).

The concentrations of IL-6 and BNP at baseline were also statistically significantly higher in the bad outcome group. Patients in the bad outcome group had worse hemodynamic parameters: lower LVEF (22.42 ± 7.19% versus 29.45 ± 9.9%, *p* = 0.005), CO (3.51 ± 1.94 ml/min versus 4.47 ± 1.26 ml/min, *p* = 0.049), CI (1.79 ± 0.80 ml/min/m^2^ versus 2.09 ± 0.71 ml/min/m^2^, *p* = 0.036), higher intracardiac pressures: mean PAP (35.5 ± 16.75 mmHg versus 24.0 ± 16.0 mmHg, *p* = 0.02), and mean PCWP (25.36 ± 9.9 mmHg versus 19.7 ± 7.56 mmHg, *p* = 0.03). Average of global strain was also significantly lower in the bad outcome group (−5.46 ± 2.3% versus −10.11 ± 2.87%, *p* = 0.001).

An increased level of circulating APN was associated with worse outcome in patients with nonischemic DCM and advanced HF.

### 3.3. Cumulative Survival Differs in Patients with High and Low Adiponectin Levels

All patients were divided into two groups according to their APN concentration at baseline: above and equal to APN median (*n* = 28) or below the median (*n* = 27). The baseline characteristics of high and low APN groups are depicted in Table 5.

Patients with APN levels above the median have also had a significantly higher BNP value (113.8 ± 694.35 pg/ml versus 1397 ± 2338.5 pg/ml, *p* < 0.001) and higher TNF-*α* concentration (7.54 ± 3.18 pg/ml versus 9.09 ± 2.54 pg/ml, *p* = 0.029). Their mean PAP (25.5 ± 13.00 mmHg versus 37.0 ± 19.0 mmHg, *p* = 0.036) was elevated in comparison to lower APN group. Cardiac output was significantly lower in the above the median APN group (3.9 ± 1.32 versus 4.9 ± 2.77, *p* = 0.035). Age, BMI, GFG, and LVEF were comparable between the groups.

**Figure 8:**
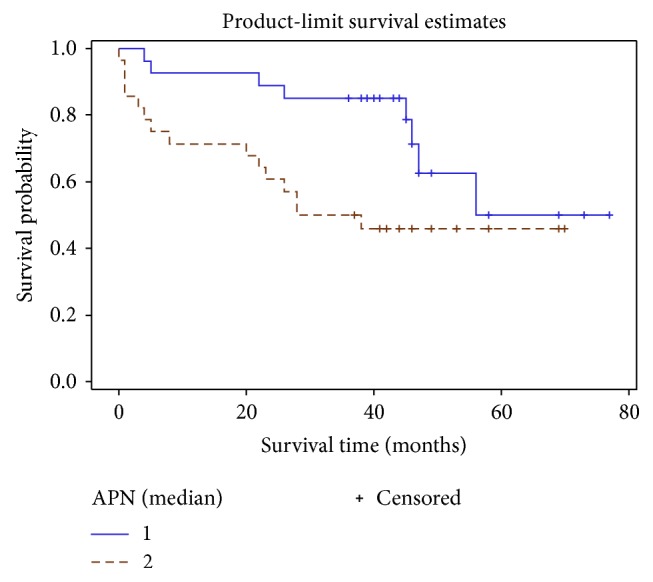
Cumulative survival curve of patient groups stratified by APN median. Straight line: APN < 14.2 ug/ml, dashed line: APN ≥ 14.2 ug/ml.

Kaplan–Meier survival curve method and log-rank analysis revealed that event-free survival times differ significantly in both groups. Worse outcome was in the higher (≥14.2 *μ*g/ml) APN group (*p* = 0.042) (Figure 8).

The biggest difference between the curves was seen for the first 4 years. Afterwards survival curves started approximating each other. So the prognostic value of APN level could be helpful in the near future.

Patients with higher APN values had a significantly increased composite endpoint risk which was the most evident in the first few years.

### 3.4. Adiponectin's and Other Parameter Role in Predicting Outcome in Patients with Nonischemic DCM and Advanced HF

Univariate Cox proportional hazard model showed that APN statistically significantly increases the risk of worse outcome (HR 1.04, *p* = 0.001). Other parameters at baseline also influenced survival and increased the risk of reaching the composite endpoint (Table 6).

NYHA functional class IV appeared to be most significantly associated with worse outcome (HR 3.84, *p* = 0.005). Average global strain also proved to be a powerful tool in predicting the risk of reaching the endpoint (HR 1.7, *p* < 0.001).

We also found that increase in IL-6 concentration (HR 1.04, *p* = 0.001), increase in number of CD3+ cells in myocardium (HR 1.06, *p* = 0.006), decrease in LVEF (HR 0.93, *p* = 0.01), and increased PAP (HR 1.06, *p* = 0.004) and PCWP (HR 1.07, *p* = 0.004) raised the risk of reaching the endpoint. What was unexpected is that BNP did not increase the risk in our model.

We looked if the impact of APN on composite endpoint remains after adjusting for other covariates in multivariate Cox regression analysis model. The covariates were age, sex, GFG, LVEF, NYHA class (which are known to have an impact on survival of patients with heart failure), and parameters which appeared to be significant in univariate regression analysis (APN, IL-6, CD 3+ cell count, and average global strain). After adjusting for these covariates, using stepwise model selection, APN lost its significance. NYHA class IV (HR 4.686, *p* = 0.012) and average global strain (HR 1.4, *p* = 0.043) appeared to be independent outcome predictors in our data (Table 7).

### 3.5. Increasing Predictive Potential of Serum Adiponectin Level in DCM Patients

Factor analysis was performed in order to see if there is a combination of parameters, which could be an expression of a more global factor having impact on patient outcome.

**Figure 9:**
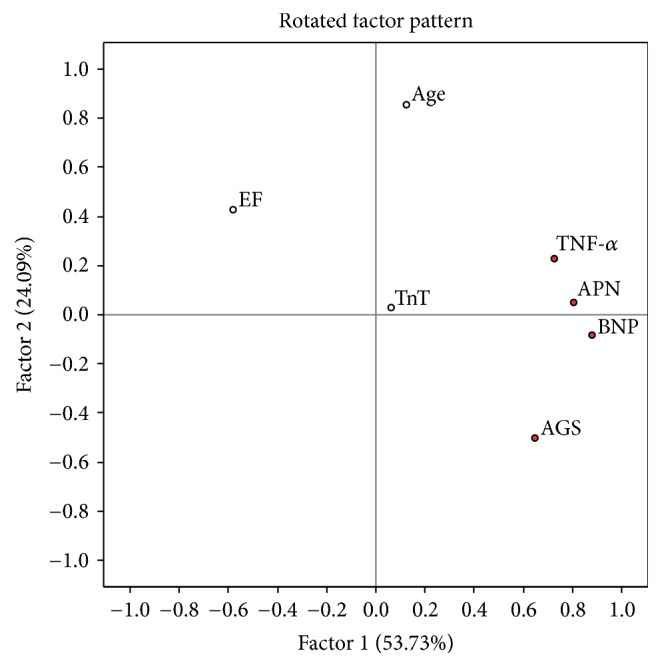
EF: left ventricle ejection fraction, TnT: troponin T, TNF-alpha: tumor necrosis factor *α*, APN: adiponectin, BNP: brain natriuretic protein, and AGS: average global strain. Parameters with maximal loadings characterizing Factor 1 are marked in red.

**Figure 10:**
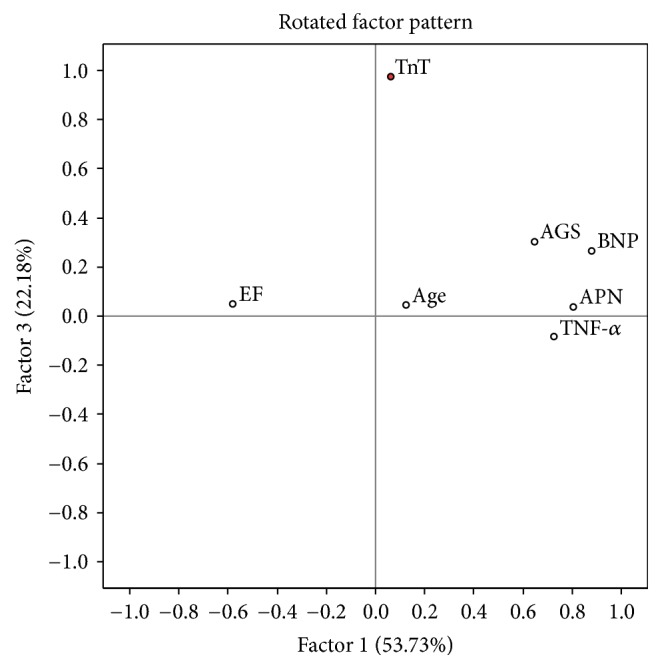
EF: left ventricle ejection fraction, TnT: troponin T, TNF-alpha: tumor necrosis factor *α*, APN: adiponectin, BNP: brain natriuretic protein, and AGS: average global strain. Parameters with maximal loadings characterizing Factor 3 are marked in red.

**Figure 11:**
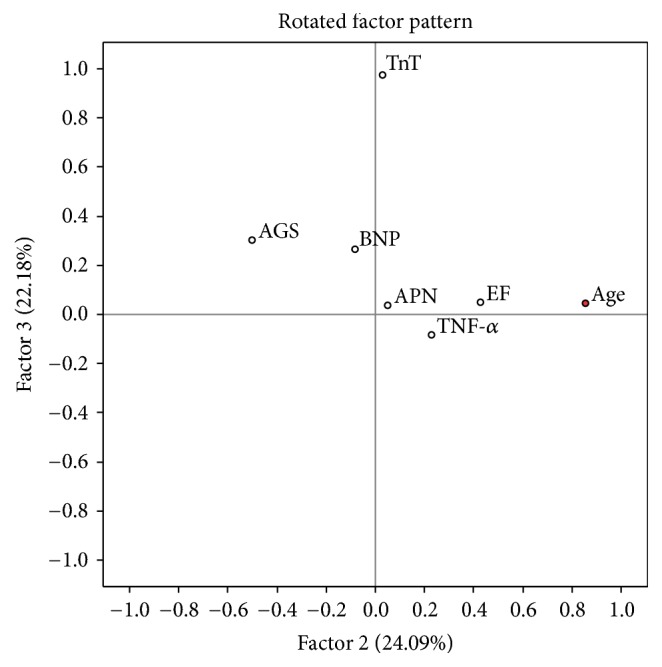
EF: left ventricle ejection fraction, TnT: troponin T, TNF-alpha: tumor necrosis factor *α*, APN: adiponectin, BNP: brain natriuretic protein, and AGS: average global strain. Parameters with maximal loadings characterizing Factor 2 are marked in red.

There were 39 cases with complete set of data which were used for the analysis. Continuous variables (age, LVEF, APN, BNP, Troponin T, TNF-*α*, and average global strain) were enrolled. Variables without normal distribution were transformed using Box-Cox method. Principal component analysis extracted 3 factors with eigenvalues above 1. The rotated factor pattern for all three factors is shown in Figures 9–11.

**Figure 12:**
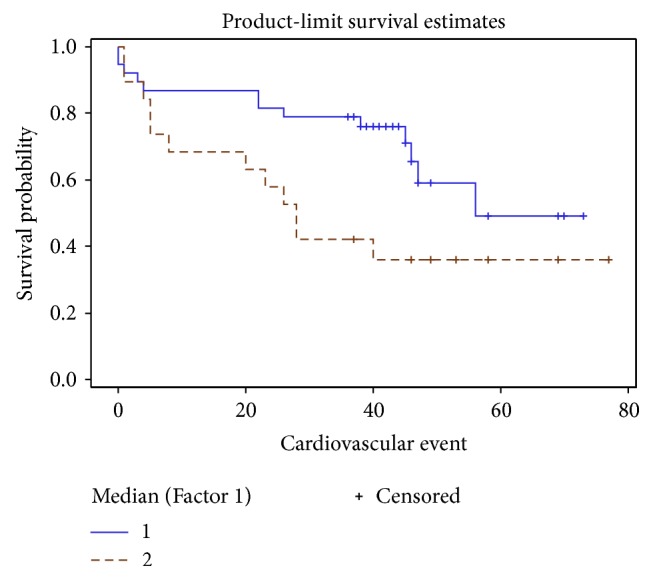
Survival curves according to Factor 1 median. Straight line: Factor 1 below the median, dashed line: Factor 1 above the median.

Factor 1 showed 53,73% of parameter variability and was characterized by a strong loading of APN, BNP, TNF-*α*, and average global strain. Those are the parameters of proinflammatory status and myocardial dysfunction. There was a significantly worse cumulative survival in Factor 1 above median group (Figure 12).

Cox regression analysis was performed for all parameters with highest Factor 1 loadings separately as well as Factor 1. Factor 1 increased the HR of composite endpoint to 2,6 (*p* = 0.0016, 95% CI 1.437–4.727) and that was more than of any individual parameter (Table 8).

Increased levels of APN and proinflammatory cytokine TNF-*α* together with the parameters of myocardial function (BNP, average global strain) could be applied in predicting patients' outcome. Combination of those parameters significantly increases APN predictive power in patients with DCM and advanced HF.

Factor 2 was best characterized by positive loading of age. Factor 3 was described mostly by troponin T, parameter of myocardial necrosis. Neither Factor 2 nor Factor 3 did show significant changes in hazard ratio or survival curves in our model.

## 4. Discussion

The purpose of this study was to investigate the predictive potential of APN with regard to LVAD implantation, HT, and mortality in a cohort of patients with nonischemic dilated DCM and advanced HF and also to analyze the association between APN and other biomarkers of CHF.

We found elevated serum APN concentrations in patients with DCM and advanced HF, similar to the ones reported by Huang et al. and Szabo et al. [35, 36]. Our findings are in agreement with previous studies [18, 23, 37]. The mechanisms of high serum APN concentration in HF are not clear; the possible reasons could be a compensatory response to HF progression or APN resistance [38, 39]. APN released from the heart may partly contribute to the increased serum APN level as reported by Takano et al. [40].

Higher circulating levels of APN are associated with increased mortality and disease severity in patients with HF [24, 40]. In our study, Kaplan–Meier survival method and log-rank analyses revealed that overall composite endpoint risk was significantly elevated in the higher serum APN group (*p* < 0.042). Univariate Cox proportional hazard model showed that increase in APN level statistically significantly elevates the risk of worse outcome (HR 1,04, *p* = 0,001), which is relevant to other publications [36]. After adjusting for other covariates (age, sex, GFG, LVEF, NYHA class, APN, IL-6, CD 3+ cell count, and average global strain) in multivariate Cox proportional hazard model APN lost its significance as independent prognostic marker. In agreement with other authors, role of APN as a predictive marker in chronic CHF is highly dependable on various clinical characteristics (age, sex, BMI, NYHA class, treatment received, renal function, type of HF, etc.) [41–44]. For this reason, clinical interpretation of APN level in patient's plasma is not straightforward and not so easily applicable in clinical practice.

Worsening of HF is associated with higher APN concentration, and increase of APN in serum parallels the increase in NYHA class [9, 21, 22, 45]. We could see the same tendency in our data concerning patients with nonischemic DCM with reduced LVEF. APN level in our cohort of patients was statistically significantly higher in NYHA class IV patients compared to NYHA class III. This observation has no easy explanation taking into account the positive functions ascribed to APN in terms of metabolism and cardioprotection. The relation of APN with HF severity confirms the fact that after implantations of LVAD the elevated APN levels have been reported to decline dramatically, in parallel with lowering of systemic and adipose-specific markers of inflammation, as well as improving insulin sensitivity [46].

Serum APN concentration is known to be related to several clinical variables. Our study confirmed correlations of adiponectin with BNP, TNF-*α*, average global strain, and increased intracardial pressures (PAP, PCWP). There was a strong positive correlation between serum APN and plasma BNP levels in our study. This could confirm the significance of the circulating APN level as a prognostic marker in patients with DCM and advanced HF. The positive relationship between the 2 molecules can be explained by the results of Tsukamoto et al. study [47] which reported that natriuretic peptides enhance the* *production of APN in human* *adipocytes in patients with advance chronic HF. In turn, recognition that natriuretic peptides stimulate APN secretion provides a mechanism linking elevated APN levels to more pronounced cardiac dysfunction and a poorer prognosis [48].

We also found that APN correlates with proinflammatory cytokine TNF-*α*. Our data do not contradict what is already known about proinflammatory state in patients with chronic HF [6, 9] which means that either APN adds up to ongoing systemic inflammation and acts as a proinflammatory factor or it is unable to overcome the increasing inflammatory milieu [26]. Unfortunately, up to now, the question remains open whether and/or when adiponectin serves as a pro- or anti-inflammatory cytokine in HF [26].

Serum APN was correlated with cardiac geometry and function according to previous studies [46, 49, 50] and was inversely associated with LVEF in elderly adjusted for BMI [51]. However, among all HF patients, there was no significant association between serum concentrations of APN and LVEF. We found significant correlations between APN and average global strain (rho 0.472, *p* = 0.002) on tissue Doppler strain measurement. Our findings are in agreement with previous studies showing that myocardial strain predicted rapid HF progression in end-stage DCM patients [52]. Myocardial strain was helpful for detecting the severity of heart failure as estimated by NYHA functional class [53]. Our data indicate that myocardial strain parameters are superior to LVEF, chamber diameter, and intracardiac pressure in predicting outcome in our patient cohort. According to our knowledge, this is the first report about the association between APN and average global strain, suggesting that serum APN could be a surrogate marker of myocardial dysfunction.

This study has some limitations which have to be pointed out. First, our study was a single center study with a small number of subjects; therefore, a study on a larger scale is warranted to confirm the relationship between worse prognosis and increased serum adiponectin levels in the iDCM patients with advanced HF. Second, the influences of drugs on the serum adiponectin levels should be considered. It is known that angiotensin-converting enzyme inhibitors, angiotensin-II receptor blockers, and *β*-blockers can improve the survival of CHF patients. All participants of our study were treated with optimal medical HF therapy when their blood samples were collected. Third, the study cohort consisted of patients with advanced HF (NYHA classes III-IV). Thus, future research is needed to confirm the validity of observed clinical correlations in patients with mild HF (NYHA I-II).

In conclusion, increased level of circulating APN was associated with higher risk of worse outcome (death from cardiovascular causes, LVAD, or HT) in nonischemic DCM patients with advanced HF. It did not appear to be an independent outcome predictor in our model. However, the combination of several sera (APN, BNP) and echocardiographic (average global strain) markers increased the outcome predicting power of APN for DCM patients. Elevated serum APN could serve as potential additive clinical prognostic marker uncovering the upcoming need to plan HT for the end-stage HF patients.

## Figures and Tables

**Table 1:** Baseline characteristics and treatment of patients.

Parameter	Value	Total number of cases
*Sex*	M: 45 (78.95%) F: 12 (21.05%)	57
*NYHA class*		55
I	1 (1.81%)	
II	5 (9.09%)	
III	38 (69.09%)	
IV	11 (20.01%)	
*iDCM*	30 (55.56%)	55
*Medications received*		
ACE inhibitors	31 (54%)	57
Diuretics and mineralocorticoids receptor blockers	55 (96%)	57
ß-Blockers	52 (91%)	57
Digitalis (in atrial fibrillation)	18 (32%)	57
Anticoagulation (atrial fibrillation, EF < 40%)	33 (58%)	57
Antiarrhythmic (class III: amiodarone)	10 (18%)	57

iDCM: inflammatory dilated cardiomyopathy, ACE: angiotensin converting enzyme.

**Table 2:** Baseline characteristics of patients.

Variable	Mean ± SD or median ± IQR^*∗*^	Total number of cases
Age (years)	47.3 ± 10.9	57
BMI (kg/m^2^)	26.84 ± 8.39^*∗*^	57
GFR (ml/min)	108.6 ± 38.6	54
Systolic BP (mmHg)	116 ± 20	57
Diastolic BP (mmHg)	80 ± 10	57
Duration of symptoms before enrollment	40 ± 53	57
Glucose (mmol/l)	5.37 ± 1.25	34
APN (*μ*g/ml)	14.2 ± 20.8^*∗*^	55
BNP (pg/ml)	727.7 ± 1796.8^*∗*^	56
Il-6 (pg/ml)	2.4 ± 4.7^*∗*^	55
TNF-*α* (pg/ml)	8.6 ± 3.37^*∗*^	55
CRP (*μ*g/ml)	4.6 ± 14.2^*∗*^	52
hsTnT (pg/ml)	29.92 ± 30.04^*∗*^	55
LVEF (%)	26.08 ± 9.5	57
LVEDD (cm)	6.8 ± 0.8	57
Average global strain (%)	−8.07 ± 3.5	41
Mean RAP (mmHg)	11 ± 6.5^*∗*^	51
Mean PAP (mmHg)	29 ± 18^*∗*^	53
Mean PCWP (mmHg)	21.8 ± 8.9	54
CO (l/min)	4.00 ± 1.88	51
CI (l/min/m^2^)	2.16 ± 1.14	51
CD3+ (cells/mm^2^)	10 ± 9^*∗*^	55
CD45ro+ (cells/mm^2^)	7 ± 5^*∗*^	55
CD68+ (cells/mm^2^)	4 ± 2	55

BMI: body mass index, GFR: glomerular filtration rate, systolic BP: systolic blood pressure, diastolic BP: diastolic blood pressure, APN: adiponectin, BNP: brain natriuretic peptide, IL-6: interleukin-6, TNF-*α*: tumor necrosis factor *α*, CRP: C reactive protein, hsTnT: high sensitivity troponin T, LVEF: left ventricular ejection fraction, LVEDD: left ventricle end diastolic diameter, RAP: right atrial pressure, PAP: pulmonary artery pressure, PCWP: pulmonary capillary wedge pressure, CD3+: T cell receptor, CD45ro+: memory T cell receptor, CD68+: monocyte/macrophage receptor, CO: cardiac output, and CI: cardiac index; ^*∗*^median ± interquartile range.

**Table 3:** APN correlations with other parameters.

Variables	Correlation coefficient	*p* value	Number of cases
BMI (kg/m^2^)	−0.266	0.054	53

Systolic BP (mmHg)	−0.061	0.66	55

Diastolic BP (mmHg)	−0.315	0.019	55

Serum glucose (mmol/l)	−0.099	0.58	33
BNP (pg/ml)	0.651	**<0.001**	54
IL-6 (pg/ml)	0.257	0.058	55
TNF-*α* (pg/ml)	0.311	**0.021**	55
LVEF (%)	−0.206	0.139	53
RAP (mmHg)	0.310	**0.030**	49
PAP (mmHg)	0.434	**0.001**	51
PCWP (mmHg)	0.388	**0.005**	51
Average global strain (%)	0.472	**0.002**	40

BMI: body mass index, systolic BP: systolic blood pressure, diastolic BP: diastolic blood pressure, BNP: B-type natriuretic peptide, IL-6: interleukin-6, TNF-*α*: tumor necrosis factor *α*, LVEF: left ventricle ejection fraction, RAP: right atrial pressure, PAP: pulmonary artery pressure, and PCWP: pulmonary capillary wedge pressure; significant correlation is bolded. Correlation is significant at the *p* level 0.05 (2-tailed).

**Table 4:** Baseline characteristics of good and bad outcome patient groups.

	Good outcome	Number of cases	Bad outcome	Number of cases	*p* value
Age (years)	49.72 ± 9.57	32	44.24 ± 11.98	25	0,08
Sex	F: 7 (22%) M: 25 (80%)	32	F: 5 (20%) M: 20 (80%)	25	0,863
NYHA functional class	I: 1 (3.3%) II: 4 (13.3%) III: 22 (73.3%) IV: 3 (10.1%)		I: 0 II: 1 (4%) III: 16 (64%) IV: 8 (32%)		0,119
Inflammatory infiltrates in myocardium	Inflammatory DCM Noninflammatory DCM	15 (51.7%) 14 (48.3%)		15 (60%) 10 (40%)	0,541
BMI (kg/m^2^)	27.96 ± 5.57	32	27.06 ± 5.17	25	0.51
GFR (ml/min)	108.4 ± 33.83	29	117.98 ± 31.38	25	0.30
Systolic BP (mmHg)	124 ± 21	32	106 ± 15	25	**0.0002**
Diastolic BP (mmHg)	80 ± 13	32	70 ± 15	25	**0.01**
Serum glucose (mmol/l)	5.45 ± 1.49	19	4.94 ± 1.25	15	0.093
APN^*∗*^ (ug/ml)	10.9 ± 17.87	32	23.4 ± 23.1	23	**0.01**
BNP^*∗*^ (pg/ml)	228 ± 915.4	31	1397.1 ± 2500.75	25	**0.004**
IL-6^*∗*^ (pg/ml)	2.01 ± 2.36	32	5.45 ± 12.29	23	**0.002**
TNF-*α*^*∗*^ (pg/ml)	8.2 ± 3.71	32	8.74 ± 4.73	23	0.239
CRP (ug/ml)	3.25 ± 15.73	28	6.55 ± 14.13	24	0.388
hsTnT^*∗*^ (pg/ml)	24.67 ± 27.8	32	32.98 ± 44.3	23	0.167
LVEDD (cm)	6.65 ± 0,67	32	7.09 ± 0.9	25	0.062
LVEF (%)	29.45 ± 9.9	32	22.56 ± 7.0	25	**0.005**
Average global strain (%)	−10.11 ± 2.87	23	−5.46 ± 2.30	18	**0.001**
Mean RAP^*∗*^ (mmHg)	9.00 ± 7.0	31	13.00 ± 15.5	20	**0.016**
Mean PAP^*∗*^ (mmHg)	24.0 ± 16.0	31	35.5 ± 16.75	22	**0.021**
Mean PCWP (mmHg)	19.7 ± 7.56	31	25.36 ± 9.9	22	**0.03**
CD3+ (cells/mm^2^)	10 ± 5	30	10 ± 11	25	0.249
CD45ro+ (cells/mm^2^)	7 ± 4	30	7 ± 7	25	0.574
CD68+ (cells/mm^2^)	5 ± 2	30	3 ± 2	25	0.716
CO (ml/min)	4.47 ± 1.26	29	3.51 ± 1.94	22	**0.049**
CI (ml/min/m^2^)	2.09 ± 0.71	30	1.79 ± 0.80	21	**0.036**

BMI: body mass index, GFR: glomerular filtration rate, systolic BP: systolic blood pressure, diastolic BP: diastolic blood pressure, APN: adiponectin, BNP: B-type natriuretic peptide, IL-6: interleukin-6, TNF-*α*: tumor necrosis factor *α*, CRP: C reactive protein, TnT: hs troponin T, LVEF: left ventricular ejection fraction, LVEDD: left ventricle diastolic diameter, RAP: right atrial pressure, PAP: pulmonary artery pressure, PCWP: pulmonary capillary wedge pressure, CD3+: T cell receptor, CD45ro+: memory T cell receptor, CD68+: monocyte/macrophage receptor, CO: cardiac output, and CI: cardiac index; significant values are bolded. Significant at the *p* level 0.05 (2-tailed). ^*∗*^Median ± interquartile range.

**Table 5:** Baseline characteristics in patient groups with high and low APN value.

Parameters	APN < 14,2 *μ*g/ml	APN ≥ 14.2 *μ*g/ml	*p* value
Sex	M: 23 (85.2%) F: 4 (14.8%)	M: 21 (75%) F: 7 (25%)	0.345
NYHA class	I + II: 4 (15.4%) III: 20 (76.9%) IV: 2 (7.7%)	I + II: 2 (7.4%) III: 16 (59.3%) IV: 9 (33.3%)	0.061
Age (years)	48.19 ± 8.29	46.96 ± 12.89	0.679
BMI^*∗*^ (kg/m^2^)	27.92 ± 5.70	24.41 ± 9.00	0.168
GFR (ml/min)	111.78 ± 27.66	110.70 ± 36.64	0.907
Systolic BP (mmHg)	121 ± 21	112 ± 20	**<0.0002**
Diastolic BP (mmHg)	75 ± 10	80 ± 10	0.647
Serum glucose (mmol/l)	5.38 ± 1.07	5.38 ± 2.39	0.913
BNP^*∗*^ (pg/ml)	113.8 ± 694.35	1397 ± 2338.5	**<0,001**
IL-6^*∗*^ (pg/ml)	2,01 ± 4.27	3,18 ± 6.08	0.245
TNF-*α*^*∗*^ (pg/ml)	7.54 ± 3.18	9.09 ± 2.54	**0.029**
CRP^*∗*^ (ug/ml)	3.05 ± 6.53	6.15 ± 13.23	0.252
TnT^*∗*^ (pg/ml)	23.55 ± 28.28	31,00 ± 31.76	0.368
LV DD (cm)	6.80 ± 0,80	6.95 ± 0.94	0.548
EF (%)	27.15 ± 9.60	26.22 ± 9.50	0.493
Average global strain (%)	−9.26 ± 3.42	−7.09 ± 3.38	0.051
CD3+^*∗*^ (cells/mm^2^)	10 ± 5	11 ± 10	0.364
CD45ro+^*∗*^ (cells/mm^2^)	6 ± 3	7 ± 5	0.493
CD68+^*∗*^ (cells/mm^2^)	5 ± 2	5 ± 2	0.769
Mean RAP^*∗*^ (mmHg)	11.00 ± 8.00	11.50 ± 11.50	0.099
Mean PAP^*∗*^ (mmHg)	25.5 ± 13.00	37.0 ± 19.00	**0.036**
Mean PCWP (mmHg)	19.50 ± 8.26	24.12 ± 8.73	0.083
CO (l/min)^*∗*^	4.9 ± 2.77	3.9 ± 1.32	**0.035**
CI (l/min/m^2^)^*∗*^	2.29 ± 1.12	2.05 ± 0.89	0.131

BMI: body mass index, GFR: glomerular filtration rate, APN: adiponectin, BNP: brain natriuretic peptide, IL-6: interleukin-6, TNF-*α*: tumor necrosis factor *α*, CRP: C reactive protein, hsTnT: high sensitivity troponin T, LVEDD: left ventricle diastolic diameter, LVEF: left ventricle ejection fraction, CD3+: T cell receptor, CD45ro+: memory T cell receptor, CD68+: monocyte/macrophage receptor, RAP: right atrial pressure, PAP: pulmonary artery pressure, PCWP: pulmonary capillary wedge pressure, CO: cardiac output, and CI: cardiac index. ^*∗*^Data presented as median ± interquartile range. Significant at the *p* level 0.05 (2-tailed).

**Table 6:** Parameters influencing outcome.

Univariate Cox regression analysis	HR	95% CI for HR	*p*
NYHA (class IV versus I–III)	3.48	1.452–8.359	**0.005**
APN (*μ*g/ml)	1.04	1.016–1.067	**0.001**
IL-6 (pg/ml)	1.04	1.01–1.07	**0.004**
BNP (pg/ml)	1.00	1.000–1.000	0.061
Average global strain (%)	1.69	1.322–2.180	**<0.001**
CD3+ (cells/mm^2^)	1.06	1.015–1.099	**0.006**
LVEF (%)	0.93	0.882–0.983	**0.010**
Mean PAP (mmHg)	1.06	1.018–1.097	**0.004**
Mean PCWP (mmHg)	1.07	1.022–1.127	**0.004**

APN: adiponectin, IL-6: interleukin-6, BNP: brain natriuretic peptide, CD3+: T cell receptor, LVEF: left ventricle ejection fraction, PAP: pulmonary artery pressure, and PCWP: pulmonary capillary wedge pressure.

**Table 7:** Independent outcome predictors.

	HR	95% CI (L) HR	95% CI (U) HR	*p* value
Average global strain	1.42	1.081	1.866	0.012
NYHA (IV class)	4.69	1.052	20.872	0.043

**Table 8:** Relative effect on patient outcome for individual parameters best characterizing Factor 1 versus Factor 1.

Parameter	HR	95% CI	*p*
APN	1.51	1.149–1.993	0.003
BNP	1.49	1.152–1.952	0.003
TNF-*α*	671.04	0.122–3698148	0.138
Average global strain	1.70	1.322–2.180	<0.001
Factor 1	2.61	1.437–4.727	0.0016

APN: transformed adiponectin value, BNP: transformed brain natriuretic peptide value, and TNF-*α*: transformed tumor necrosis factor *α* value.
